# Chewing increases postprandial diet-induced thermogenesis

**DOI:** 10.1038/s41598-021-03109-x

**Published:** 2021-12-09

**Authors:** Yuka Hamada, Naoyuki Hayashi

**Affiliations:** 1 Department of Nutrition and Metabolism, National Institute of Health and Nutrition, National Institutes of Biomedical Innovation, Health and Nutrition, 1-23-1 Toyama, Shinjuku-ku, Tokyo, 162-8636 Japan; 2 Waseda Institute for Sport Sciences, Waseda University, 2-579-15 Mikajima, Tokorozawa, Saitama 359-1192 Japan; 3 Faculty of Sport Sciences, Waseda University, 2-579-15 Mikajima, Tokorozawa, Saitama 359-1192 Japan; 4 Institute for Liberal Arts, Tokyo Institute of Technology, Ookayama 2-12-1, Meguro, 152-8552 Japan

**Keywords:** Metabolism, Nutrition, Weight management

## Abstract

Slow eating, which involves chewing food slowly and thoroughly, is an effective strategy for controlling appetite in order to avoid being overweight or obese. Slow eating also has the effect of increasing postprandial energy expenditure (diet-induced thermogenesis). It is still unclear whether this is due to oral stimuli; that is, the duration of tasting food in the mouth and the duration of chewing. To investigate the effects of oral stimuli on diet-induced thermogenesis in 11 healthy normal weight males, we conducted a randomized crossover study comprising three trials: (1) drinking liquid food normally, (2) drinking liquid food after tasting, and (3) adding chewing while tasting. Oral stimuli (i.e., the duration of tasting liquid food in the mouth and the duration of chewing) significantly increased diet-induced thermogenesis after drinking liquid food. This result demonstrates that the increase in diet-induced thermogenesis is due to oral stimuli rather than the influence of the food bolus. Increased diet-induced thermogenesis induced by chewing and taste stimuli may help to prevent overweight and obesity.

## Introduction

A century ago, Horce Fletcher (1849–1919), who was nicknamed “the great masticator”, found that the strategy of chewing food thoroughly could prevent weight gain, and he reported his strategy worldwide^1,2^. Many subsequent studies have indicated that slow eating, which involves chewing food slowly and thoroughly, is an effective strategy for preventing overweight and obesity, with eating speed being associated with body composition and shape^3–18^. This has been attributed to overeating resulting from rapid eating^19,20^.

Slow eating also has the effect of increasing diet-induced thermogenesis (DIT), as we have reported previously^21,22^. DIT, which is also called the thermic effect or a specific dynamic action of food consumption, can be defined as the increase in the energy expenditure above the basal fasting level associated with the digestion, absorption, transport, metabolism, and storage of food^23^. This factor is considered to represent an increase in metabolism, in particular in brown adipose tissue, induced by the increase in histamine secretion accompanying taste stimulation and chewing for a longer time^24–27^. Increased intestinal motility could be another contributing factor, since the blood flow response increases after slow eating^21,22^. However, previous investigations of the influence of chewing on DIT have been confounded by the size of the food bolus entering the digestive tract.

The effects of oral stimuli (i.e., the duration of tasting food in the mouth and the duration of chewing) on DIT have not been reported previously. It is important to examine the effects of these oral stimuli on DIT in order to clarify the effect of slow eating as one of the strategies for preventing overweight and obesity. We hypothesized that these oral stimuli increase DIT based on our previous findings that slow eating increased DIT. We performed three trials in the present study: (1) drinking liquid food normally, (2) drinking liquid food after tasting, and (3) adding chewing while tasting to exclude the effect of the food bolus.

## Methods

### Subjects

Eleven healthy normal-weight males [age, 23 ± 1 years (mean ± SD); height, 176 ± 4 cm; body mass, 68 ± 5 kg; body mass index (BMI), 21.9 ± 1.5 kg/m^2^] participated in the present study. The inclusion criteria for this study were male, aged 18–30 years, have BMI of 18–25 kg/m^2^, non-smoker, no acute or chronic disease, no dental problems, not taking any medications, free from food allergies. This study was conducted according to the guidelines laid down in the Declaration of Helsinki and approved by the Ethics Committee of Tokyo Institute of Technology, Japan (approval number: A14095). Each subject provided written informed consent to participate prior to the commencement of the study.

### Protocol

**Figure 1:**
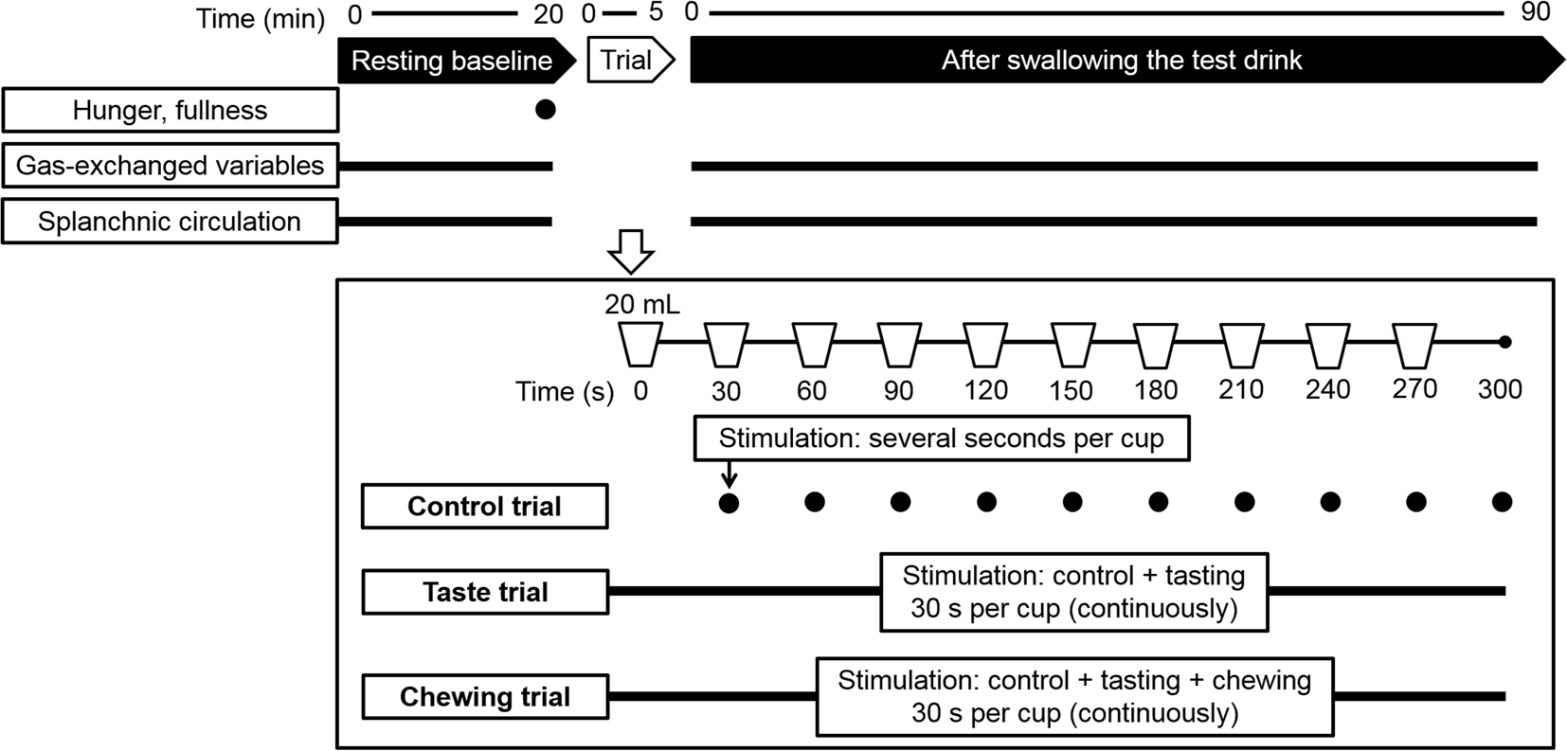
Schematic of the study protocol.

The study had a randomized crossover design (Fig. 1). The subjects completed three trials on three different days, with consecutive trial separated by more than 3 days. The subjects arrived at the laboratory at the same time between 8:30 a.m. and 10:00 a.m. after having abstained from eating, consuming caffeinated or alcoholic beverages, and intense exercise since dinner on the previous night, i.e., they had fasted for more than 10 h. Each subject was seated on a chair in a semisupine position in a quiet room in which the temperature and humidity were controlled to within 25.4 ± 0.4 °C and 47 ± 7%, respectively. After allowing the subjects to adjust to the experimental setup for 20 min, baseline data of gas-exchange variables and the splanchnic circulation were recorded while resting for 20 min. The subjects completed a 100-mm visual analogue scale (VAS) questionnaire to assess their hunger and fullness before the test drink. A 200-mL cocoa-flavored drink was divided into ten 20-mL cups. After performing baseline measurements, the subjects swallowed the ten 20-mL test drinks over a 5-min experimental period in three ways. In the control trial, subjects swallowed one 20-mL test drink every 30 s. In the long-duration taste stimulation trial (taste trial), subjects kept the 20-mL test drink in their mouth for 30 s without chewing, and then swallowed it; stimulation was taste only. In the chewing stimulation trial (chewing trial), subjects chewed the 20-mL test drink for 30 s at a frequency of once per second, and then swallowed it; stimulation was both taste and chewing. Gas-exchange variables and the splanchnic circulation were measured until 90 min after swallowing the test drink.

### Test drink

The subjects consumed the same 200-mL cocoa-flavored drink (Calorie Mate Can, Otsuka, Japan; 200 kcal; protein 7.6 g, fat 4.4 g, and carbohydrate 31 g) as the test drink at the three trials. The temperature of the test drink was measured using an infrared thermometer (A&D, Japan), and the test drink was provided at a controlled temperature (7.4 ± 0.5 °C).

### Hunger and fullness

Subjects scored their hunger and fullness on a 100-mm VAS before the test drink. The subjects marked a tick on the line to indicate their feeling, with the score corresponding to the distance in millimeters from the left starting point of the line to the tick. The left and right ends of the scale were labelled “not at all” and “extremely”, respectively.

### Gas exchanged variables and DIT

Oxygen uptake (·VO_2_), carbon dioxide output (·VCO_2_), and respiratory exchange ratio (RER) were measured using a gas analyzer (AE-310S, Minato Medical Science, Japan) on a breath-by-breath basis before and after the test drink. The average data every 15 min and the last 15 min of the resting baseline were used for the analysis. Energy expenditure at resting baseline (REE) and after the test drink was calculated using the abbreviated Weir equation^28^:

$${\text{Energy expenditure }}\left( {{\text{EE}},{\text{ kcal}}/{\text{min}}} \right) \, = { 3}.{9 } \times {\dot{\text{V}}\text{O}}_{{2}} \left( {{\text{L}}/{\text{min}}} \right) \, + { 1}.{1 } \times {\dot{\text{V}}\text{CO}}_{{2}} \left( {{\text{L}}/{\text{min}}} \right).$$

DIT was calculated from the postprandial increments in energy expenditure above the resting baseline. Accumulation for DIT over 90 min was calculated area under the curve (AUC) using the trapezoidal rule.

### Substrate oxidation

Substrate oxidation was calculated from ·VO_2_ and ·VCO_2_ using the following formulas^29^:

$${\text{Protein oxidation }}\left( {{\text{g}}/{\text{min}}} \right) \, = \, \left[ {{\text{resting energy expenditure }}\left( {{\text{REE}},{\text{ kcal}}/{\text{min}}} \right) \, \times \, 0.{152}} \right] \, /{\text{ 4 kcal}}$$

$${\text{Fat oxidation }}\left( {{\text{g}}/{\text{min}}} \right) \, = { 1}.{67 } \times {\dot{\text{V}}\text{O}}_{{2}} \left( {{\text{L}}/{\text{min}}} \right) \, {-}{ 1}.{67 } \times {\dot{\text{V}}\text{CO}}_{{2}} \left( {{\text{L}}/{\text{min}}} \right) \, {-} \, 0.{3}0{7 } \times {\text{ protein oxidation }}\left( {{\text{g}}/{\text{min}}} \right)$$

$${\text{Carbohydrate oxidation }}\left( {{\text{g}}/{\text{min}}} \right) \, = { 4}.{55 } \times {\dot{\text{V}}\text{CO}}_{{2}} \left( {{\text{L}}/{\text{min}}} \right) \, {-}{ 3}.{21 } \times {\dot{\text{V}}\text{O}}_{{2}} \left( {{\text{L}}/{\text{min}}} \right) \, {-} \, 0.{459 } \times {\text{ protein oxidation }}\left( {{\text{g}}/{\text{min}}} \right).$$

Protein oxidation was assumed to be 15.2% of total REE, as 15.2% energy balance of protein contained in the test drink. Accumulation for substrate oxidation over 90 min was calculated from AUC using the trapezoidal rule.

### Splanchnic circulation

The heart rate (HR) and the mean blood velocities (MBVs) and vessel diameters of the celiac artery (CA) and superior mesenteric artery (SMA) were measured. HR was determined using the electrocardiograph (MEG-2000, Nihon Kohden, Japan). Simultaneous pulsed and echo Doppler ultrasound flowmetry was used to measure the MBVs and vessel diameters of the CA and SMA, as in previous studies of our research group^21,22,30,31^. A curved-array Doppler-scan probe was operated at a pulsed Doppler frequency of 3.3 MHz (LOGIQ P6, GE Healthcare, USA), and the Doppler beam insonation angle relative to the blood vessel was maintained at ≤ 60°. After obtaining these signals for measuring MBV for 1 min, a cross-sectional image of the vessel was recorded for 30 s. This was repeated every 5 min. The B-mode images sent from the Doppler monitor were recorded to enable later measurement of the vessel diameters using image-editing software (ImageJ 1.47, Wayne Rasband, National Institute of Mental Health, USA). The HR, MBVs and EMG signals of the chewing muscles were sampled at 20 kHz using an A/D converter (PowerLab 8/30, ADInstruments, Australia). The spectra of the MBV signals were analyzed offline with our own Doppler signal processing software, and beat-by-beat MBV values were calculated. MBV was determined by averaging the ten largest values in each minute (for 1 min every 5 min) in order to eliminate data variations originating from the abdominal movement associated with respiration^30,31^. The blood flows (BFs) in the CA and SMA were calculated using the following formula:

$${\text{Blood flow }}\left( {{\text{mL}}/{\text{min}}} \right)\, = \,\pi \, \times \,{\text{r }}\left[ {{\text{radius of artery }}\left( {{\text{mm}}} \right)} \right]^{{2}} \, \times \,{\text{MBV}}\left( {{\text{m}}/{\text{s}}} \right)\, \times \,{6}0.$$

Accumulation for splanchnic BF in the CA and SMA over 90 min was calculated from incremental AUC using the trapezoidal rule.

### Statistical analysis

The sample size was estimated using G*Power 3.1^32^, using the data from a previous study that investigated the effects of the number of chews and meal duration on DIT^22^. To detect changing of DIT with a power of 80% and an alpha level of 5%, a sample size of more than six subjects was required. All statistical analyses were performed with SPSS (IBM SPSS Statistics 21.0, IBM, Japan). A *P* value of less than 0.05 was considered statistically significant, and the data were presented as mean ± SEM (range) values. One-way analysis of variance (ANOVA) was used to compare hunger and fullness scores before the test drink, baseline data of gas-exchange variables, substrate oxidation, and splanchnic circulation, and accumulation for DIT, substrate oxidation, and splanchnic BF over 90 min after consuming the test drink among all trials. Two-way repeated ANOVA was used to examine effects of trials and time on time course data. When a significant *F* value was detected, this was further examined by using Bonferroni’s post-hoc test.

## Results

### Hunger and fullness

At the resting baseline, the hunger and fullness scores did not differ significantly among the trials: hunger [mean ± SEM (range)], 74 ± 6 (30–99) mm, 76 ± 4 (52–93) mm, 81 ± 3 (66–94) mm; fullness, 16 ± 3 (1–35), 18 ± 4 (5–38), 13 ± 4 (1–36) in the control, taste, and chewing trials, respectively. There was no difference among trials after the intake.

### Gas-exchange variables, DIT, and substrate oxidation

**Figure 2:**
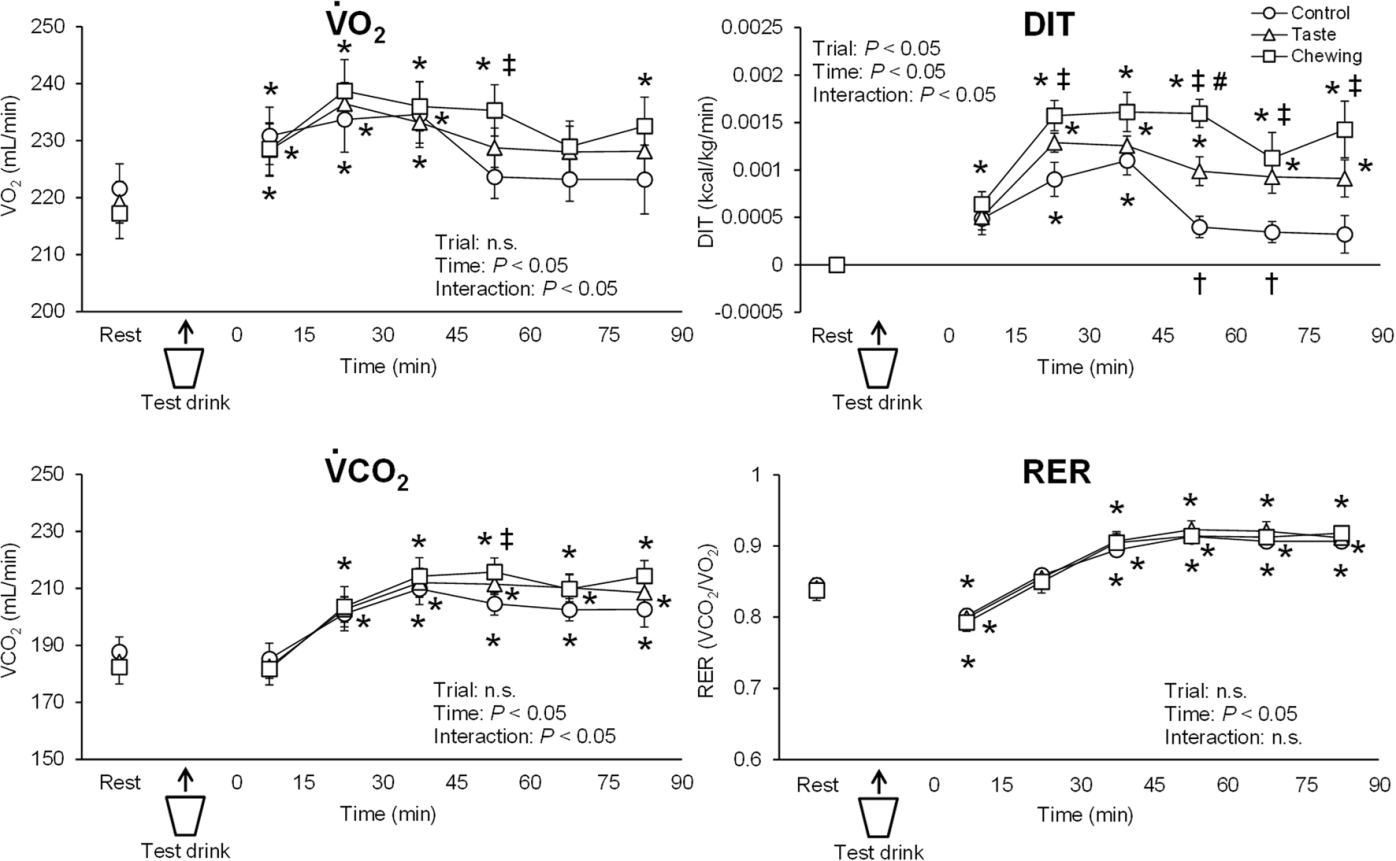
Time courses of changes in gas-exchange variables and diet-induced thermogenesis (DIT) in the control, taste, and chewing trials. Circles, triangles, and squares denote data from the control, taste, and chewing trials, respectively. ·*VO*_*2*_ oxygen uptake, *DIT* diet-induced thermogenesis, *·VCO*_*2*_ carbon dioxide output, *RER* respiratory exchange ratio. ^*^P < 0.05, vs. resting baseline in each trial. ^†^*P* < 0.05, difference between control and taste trials. ^‡^*P* < 0.05, difference between control and chewing trials. ^#^*P* < 0.05, difference between taste and chewing trials.

The time courses of the gas-exchange variables (·VO_2_, ·VCO_2_, and RER) are shown in Fig. 2. The gas-exchange variables at the resting baseline did not differ significantly among the trials. Significant interaction of trial and time was found for ·VO_2_. ·VO_2_ was significantly greater in the chewing trial than in the control trial at 45–60 min after the test drink. The duration over which ·VO_2_ was significantly greater than the resting baseline was longer in the chewing trial than in the control and taste trials. Significant interaction of trial and time was found for ·VCO_2_. ·VCO_2_ was significantly greater in the chewing trial than in the control trial at 45–60 min after the test drink. RER did not differ significantly among the trials.

A significant interaction of trial and time was found for DIT (Fig. 2). DIT was significantly greater in the taste and chewing trials than in the control trial; these differences continued until 90 min after the test drink between chewing and control trials, and was evident at 45–75 min between taste and control trials. The duration over which DIT was significantly greater than the resting baseline was longer in the taste and chewing trials than in the control trial.

**Figure 3:**
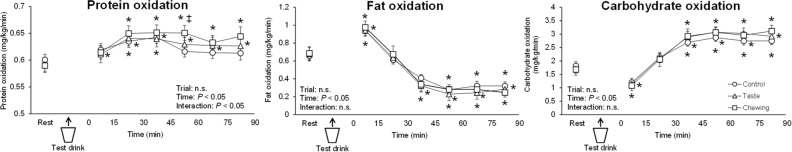
Time courses of changes in substrate oxidation (protein, fat, and carbohydrate) in the control, taste, and chewing trials. Circles, triangles, and squares denote data from the control, taste, and chewing trials, respectively. ^*^P < 0.05, vs. resting baseline in each trial. ^‡^*P* < 0.05, difference between control and chewing trials.

The time courses of the substrate oxidations (protein, fat, and carbohydrate) are shown in Fig. 3. Significant interaction of trial and time was found for protein oxidation. Protein oxidation was significantly greater in the chewing trial than in the control trial at 45–60 min after the test drink. Fat and carbohydrate oxidation did not differ significantly among the trials.

### Splanchnic circulation

**Figure 4:**
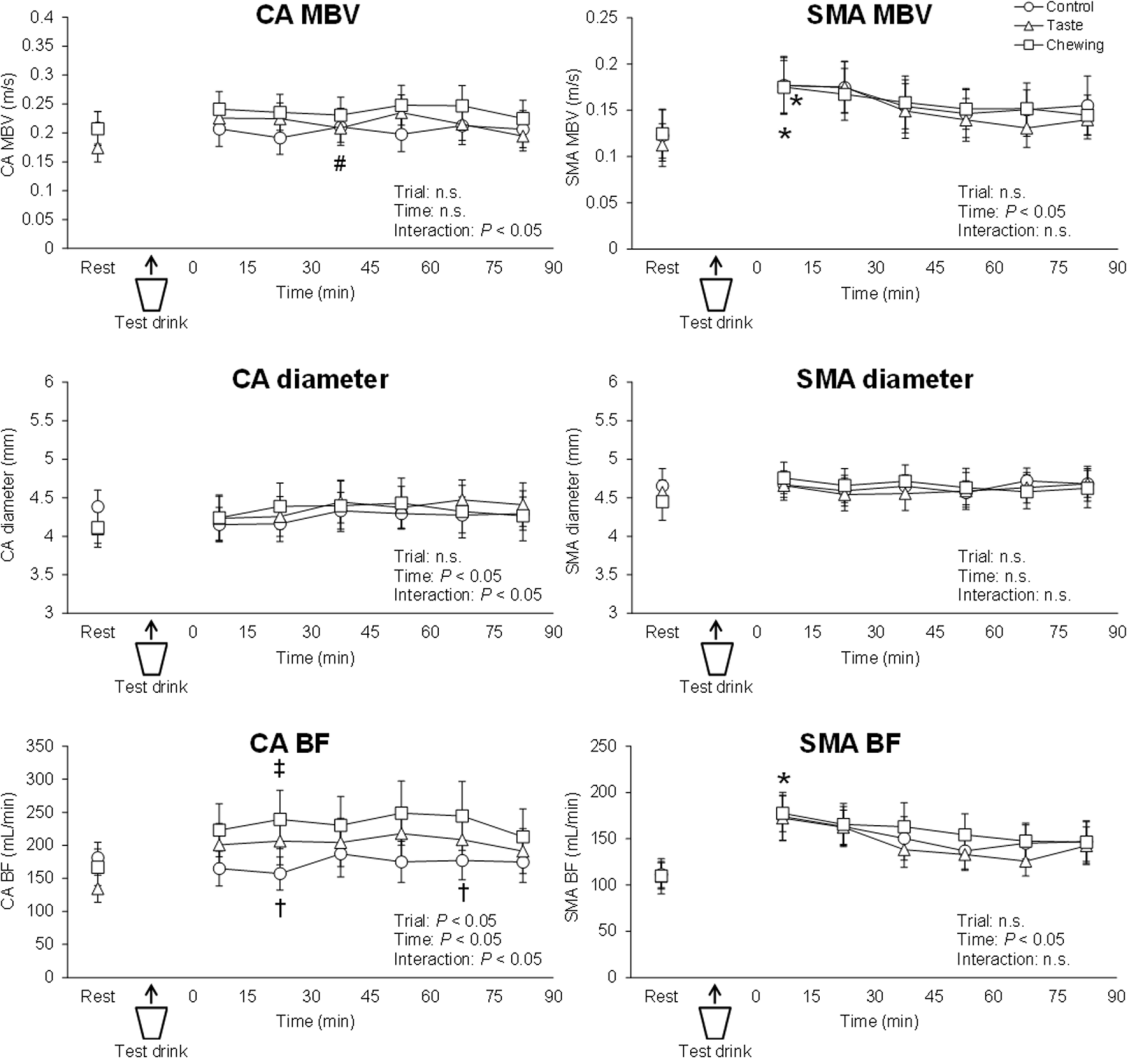
Time courses of changes in splanchnic circulation in the control, taste, and chewing trials. Circles, triangles, and squares denote data from the control, taste, and chewing trials, respectively. *CA* celiac artery, *SMA* superior mesenteric artery, *MBV* mean blood velocity, *BF* blood flow. ^*^P < 0.05, vs. resting baseline in each trial. ^†^*P* < 0.05, difference between control and taste trials. ^‡^*P* < 0.05, difference between control and chewing trials. ^#^*P* < 0.05, difference between taste and chewing trials.

The time courses of the splanchnic circulation (MBV, diameter, BF for the CA and SMA) are shown in Fig. 4. The splanchnic circulation at the resting baseline did not differ significantly among the trials. Significant interaction of trial and time was found for CA MBV. CA MBV was significantly greater in the chewing trial than in the taste trial at 30–45 min after the test drink. Significant interaction of trial and time was found for CA diameter. CA diameter did not differ significantly among the trials. Significant interaction of trial and time was found for CA BF. CA BF was significantly greater in the taste and chewing trials than in the control trial; these differences was evident at 15–30 min and 60–75 min between taste and control trials, and was evident at 15–30 min between chewing and control trials. SMA MBV, SMA diameter, and SMA BF did not differ significantly among the trials.

### Accumulated variables over the 90 min after the test drink

**Figure 5:**
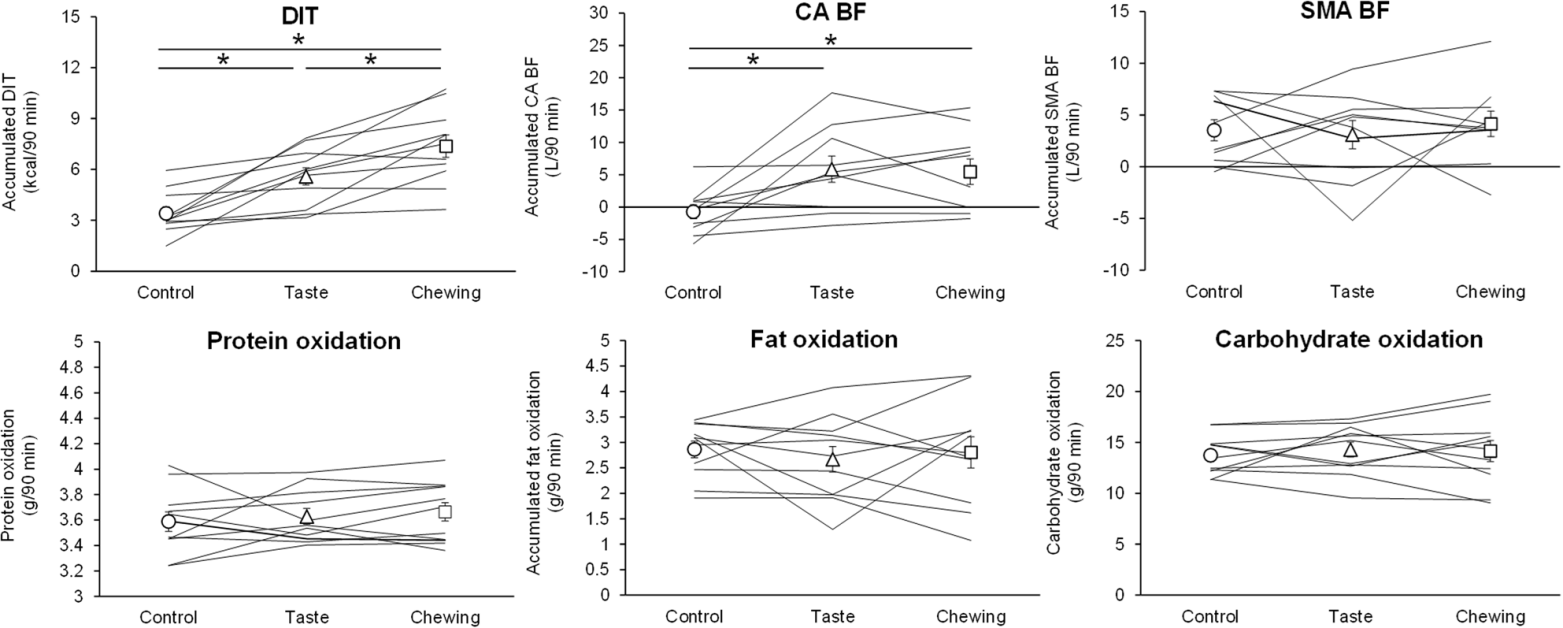
Diet-induced thermogenesis (DIT), substrate oxidation, and postprandial splanchnic circulation accumulated over the 90-min period immediately after consuming the test drink. Lines join individual values, and symbols (circles, triangles, and squares) indicate mean values. *DIT* diet-induced thermogenesis, *CA* celiac artery, *SMA* superior mesenteric artery, *BF* blood flow. ^*^P < 0.05, difference between trials.

The DIT that accumulated over the 90 min after swallowing the test drink was significantly greater in the chewing trial than in the control and taste trials [control trial, 3.4 ± 0.4 (1.5–5.9) kcal; taste trial, 5.6 ± 0.5 (3.2–7.8) kcal; chewing trial, 7.4 ± 0.7 (3.6–10.7) kcal] (Fig. 5). The accumulated substrate oxidation for protein, fat, and carbohydrate did not differ significantly among trials [protein oxidation: control trial, 3.6 ± 0.1 (3.2–4.0) g; taste trial, 3.6 ± 0.1 (3.4–4.0) g; chewing trial, 3.7 ± 0.1 (3.4–4.1) g, fat oxidation: control trial, 2.9 ± 0.2 (1.9–3.4) g; taste trial, 2.7 ± 0.3 (1.3–4.1) g; chewing trial, 2.8 ± 0.3 (1.1–4.3) g, carbohydrate oxidation: control trial, 13.7 ± 0.6 (11.3–16.7) g; taste trial, 14.3 ± 0.7 (9.5–17.3) g; chewing trial, 14.2 ± 1.0 (9.1–19.7) g]. The accumulated CA BF was significantly greater in the taste and chewing trial than in the control trial [control trial, -0.7 ± 1.1 (-5.7–6.2) L; taste trial, 5.9 ± 2.0 (-2.8–17.7) L; chewing trial, 5.5 ± 2.0 (-1.8–15.4) L], while the accumulated SMA BF did not differ significantly among trials [control trial, 3.5 ± 1.0 (-0.5–7.3) L; taste trial, 3.1 ± 1.4 (-5.2–9.4) L; chewing trial, 4.1 ± 1.2 (-2.7–12.1) L].

## Discussion

DIT refers to the increased energy production after consuming a meal, and it was found to increase with the duration of each taste stimulation and the duration of chewing. This result, supporting our hypothesis, demonstrates that oral stimuli (i.e., the duration of tasting food in the mouth and the duration of chewing) increase DIT, rather than representing the influence of the food bolus. CA BF increased with the duration of each of taste stimulation and with the duration of chewing. CA supplies blood to the liver, stomach, abdominal esophagus, spleen, and the superior halves of the duodenum and the pancreas, and so the motility of the upper gastrointestinal tract was increased by taste and chewing. This notion is consistent with our previous report of an increase in CA BF associated solely with taste and chewing food^30^.

In this study we used a beverage as the stimulus in order to avoid the influence of the food bolus, which meant that we were only examining the influence of oral stimuli on DIT. We have previously shown using blocky foods and regular foods that DIT increases with the chewing duration^21,22^, but did not control for the effect of the food bolus. By using beverages, we succeeded in eliminating this effect in the present study.

The present study provides novel insight into the mechanism underlying the increase in DIT induced by taste and chewing. We have also provided evidence that the oral stimuli provided by the combination of taste and chewing are important to increasing DIT. Thus, slow eating, which involves chewing food slowly and thoroughly, increases DIT and may be an effective strategy for preventing overweight and obesity.

The increase in DIT induced by chewing is smaller after consuming solely liquid food than normal food. We have previously shown that chewing increases DIT: by 6 kcal and 10 kcal over 90 min after eating 100-kcal and 300-kcal blocky foods, respectively, and by 15 kcal over 180 min after eating 621 kcal of regular food including pasta^21,22^. Comparison with these previous results reveals that we obtained a smaller DIT by using a 200-kcal beverage: tasting increased DIT from 3.4 kcal to 5.6 kcal over 90 min, and adding chewing increased it to 7.4 kcal over 90 min. Thus, the increase in DIT by chewing was 1.8–4.0 kcal over 90 min. The use of liquid reduces the chewing stimulation, resulting in a smaller DIT, which is consistent with our hypothesis. This reveals that not only oral stimuli (i.e., the duration of tasting food in the mouth and the duration of chewing) but also the size of the food bolus may contribute to DIT.

## Conclusion

Oral stimuli (i.e., the duration of tasting food in the mouth and the duration of chewing) increase DIT. We speculate that overweight and obesity may be avoided by chewing and tasting via increases in DIT.

## References

[1] Smit HJ, Kemsley EK, Tapp HS, Henry CJ (2011). Does prolonged chewing reduce food intake? Fletcherism revisited. Appetite.

[2] Christen AG, Christen JA (1997). Horace Fletcher (1849-1919): "The great masticator". J. Hist. Dent..

[3] Sonoda C (2018). Associations among obesity, eating speed, and oral health. Obes. Facts.

[4] Tao L (2018). Association between self-reported eating speed and metabolic syndrome in a Beijing adult population: A cross-sectional study. BMC Public Health.

[5] van den Boer JHW (2017). Self-reported eating rate is associated with weight status in a Dutch population: A validation study and a cross-sectional study. Int. J. Behav. Nutr. Phys. Act..

[6] Hamada Y (2017). Objective and subjective eating speeds are related to body composition and shape in female college students. J. Nutr. Sci. Vitaminol..

[7] Zhu B, Haruyama Y, Muto T, Yamazaki T (2015). Association between eating speed and metabolic syndrome in a three-year population-based cohort study. J. Epidemiol..

[8] Ohkuma T (2015). Association between eating rate and obesity: A systematic review and meta-analysis. Int. J. Obes. (Lond.).

[9] Yamane M (2014). Relationships between eating quickly and weight gain in Japanese university students: A longitudinal study. Obesity (Silver Spring).

[10] Nagahama S (2014). Self-reported eating rate and metabolic syndrome in Japanese people: Cross-sectional study. BMJ Open.

[11] Ochiai H (2013). Eating behaviors and overweight among adolescents: A population-based survey in Japan. J. Obes..

[12] Ekuni D, Furuta M, Takeuchi N, Tomofuji T, Morita M (2012). Self-reports of eating quickly are related to a decreased number of chews until first swallow, total number of chews, and total duration of chewing in young people. Arch. Oral. Biol..

[13] Sakurai M (2012). Self-reported speed of eating and 7-year risk of type 2 diabetes mellitus in middle-aged Japanese men. Metabolism.

[14] Tanihara S (2011). Retrospective longitudinal study on the relationship between 8-year weight change and current eating speed. Appetite.

[15] Leong SL, Madden C, Gray A, Waters D, Horwath C (2011). Faster self-reported speed of eating is related to higher body mass index in a nationwide survey of middle-aged women. J. Am. Diet Assoc..

[16] Maruyama K (2008). The joint impact on being overweight of self reported behaviours of eating quickly and eating until full: Cross sectional survey. BMJ.

[17] Otsuka R (2006). Eating fast leads to obesity: Findings based on self-administered questionnaires among middle-aged Japanese men and women. J. Epidemiol..

[18] Sasaki S, Katagiri A, Tsuji T, Shimoda T, Amano K (2003). Self-reported rate of eating correlates with body mass index in 18-y-old Japanese women. Int. J. Obes. Relat. Metab. Disord..

[19] Robinson E (2014). A systematic review and meta-analysis examining the effect of eating rate on energy intake and hunger. Am. J. Clin. Nutr..

[20] Andrade AM, Greene GW, Melanson KJ (2008). Eating slowly led to decreases in energy intake within meals in healthy women. J. Am. Diet Assoc..

[21] Hamada Y, Miyaji A, Hayashi N (2016). Effect of postprandial gum chewing on diet-induced thermogenesis. Obesity.

[22] Hamada Y, Kashima H, Hayashi N (2014). The number of chews and meal duration affect diet-induced thermogenesis and splanchnic circulation. Obesity (Silver Spring).

[23] Levine JA (2004). Non-exercise activity thermogenesis (NEAT). Nutr. Rev..

[24] Sakata T, Yoshimatsu H, Masaki T, Tsuda K (2003). Anti-obesity actions of mastication driven by histamine neurons in rats. Exp. Biol. Med. (Maywood).

[25] Masaki T, Yoshimatsu H, Chiba S, Watanabe T, Sakata T (2001). Targeted disruption of histamine H1-receptor attenuates regulatory effects of leptin on feeding, adiposity, and UCP family in mice. Diabetes.

[26] Sakata T (1995). Histamine receptor and its regulation of energy metabolism. Obes. Res..

[27] Fujise T (1993). Food consistency modulates eating volume and speed through brain histamine in rat. Brain Res. Bull..

[28] Weir JB (1949). New methods for calculating metabolic rate with special reference to protein metabolism. J. Physiol..

[29] Jéquier E, Felber JP (1987). Indirect calorimetry. *Baillieres*. Clin. Endocrinol. Metab..

[30] Someya N, Hayashi N (2008). Chewing and taste increase blood velocity in the celiac but not the superior mesenteric arteries. Am. J. Physiol. Regul. Integr. Comp. Physiol..

[31] Someya N, Endo MY, Fukuba Y, Hayashi N (2008). Blood flow responses in celiac and superior mesenteric arteries in the initial phase of digestion. Am. J. Physiol. Regul. Integr. Comp. Physiol..

[32] Faul F, Erdfelder E, Lang AG, Buchner A (2007). G*Power 3: a flexible statistical power analysis program for the social, behavioral, and biomedical sciences. Behav. Res. Methods.

