# Publisher Correction: Chewing increases postprandial diet-induced thermogenesis

**DOI:** 10.1038/s41598-021-04257-w

**Published:** 2021-12-23

**Authors:** Yuka Hamada, Naoyuki Hayashi

**Affiliations:** 1 Department of Nutrition and Metabolism, National Institute of Health and Nutrition, National Institutes of Biomedical Innovation, Health and Nutrition, 1-23-1 Toyama, Shinjuku-ku, Tokyo, 162-8636 Japan; 2 Waseda Institute for Sport Sciences, Waseda University, 2-579-15 Mikajima, Tokorozawa, Saitama 359-1192 Japan; 3 Faculty of Sport Sciences, Waseda University, 2-579-15 Mikajima, Tokorozawa, Saitama 359-1192 Japan; 4 Institute for Liberal Arts, Tokyo Institute of Technology, Ookayama 2-12-1, Meguro, 152-8552 Japan

Correction to: *Scientific Reports* 10.1038/s41598-021-03109-x, published online 09 December 2021

The original version of this Article omitted an affiliation for Naoyuki Hayashi. The correct affiliations are listed below.

Faculty of Sport Sciences, Waseda University, 2-579-15 Mikajima, Tokorozawa, Saitama, 359-1192, Japan

Institute for Liberal Arts, Tokyo Institute of Technology, Ookayama 2-12-1, Meguro 152-8552, Japan.

The original Article has been corrected.

